# Extracellular water to total body water ratio predicts survival in cancer patients with sarcopenia: a multi-center cohort study

**DOI:** 10.1186/s12986-022-00667-3

**Published:** 2022-05-07

**Authors:** Yi-Zhong Ge, Guo-Tian Ruan, Qi Zhang, Wen-Jun Dong, Xi Zhang, Meng-Meng Song, Xiao-Wei Zhang, Xiang-Rui Li, Kang-Ping Zhang, Meng Tang, Wei Li, Xian Shen, Han-Ping Shi, Investigation on the Nutrition Status and Clinical Outcome of Common Cancers (INSCOC) Group

**Affiliations:** 1 Department of Gastrointestinal Surgery/Department of Clinical Nutrition, Beijing Shijitan Hospital, Capital Medical University, Beijing, 100038 China; 2 The Second Affiliated Hospital and Yuying Children’s Hospital of Wenzhou Medical University, Wenzhou, 325000 China; 3 Beijing International Science and Technology Cooperation Base for Cancer Metabolism and Nutrition, Beijing, 100038 China; 4 Key Laboratory of Cancer FSMP for State Market Regulation, Beijing, 100038 China; 5 Cancer Center of the First Hospital of Jilin University, Changchun, 130021 Jilin China

**Keywords:** Body water, Extracellular water, Cancer sarcopenia, Bioelectrical impedance analysis

## Abstract

**Background:**

Body water measured by bioelectrical impedance analysis (BIA) predicts the outcomes of many diseases. This study aimed to evaluate the relationship between body water and the prognosis of cancer patients with sarcopenia.

**Methods:**

This study employed 287 cancer patients with sarcopenia underwent BIA from a prospective multicenter study of patients with cancer in China from 2013 to 2020. The primary outcome of interest was all-cause mortality presented as the longest time to follow-up available. Eight indicators of body water [total body water, extracellular water, intracellular water, free fat mass, active cell mass, extracellular water/intracellular water, extracellular water/total body water (ECW/TBW), and intracellular water/total body water] were included in the research. Neutrophil–lymphocyte ratio (NLR) = neutrophil (× 10^9^)/lymphocyte (× 10^9^). The discriminatory ability and prediction accuracy of each factor were assessed using the C-index. The hazard ratios (HR) and 95% confidence intervals (CI) were calculated using the Cox proportional hazard model.

**Results:**

The median age was 65 years old, and 138 (48%) patients were men. During a mean follow-up of 46 months, 140 deaths were recorded, resulting in a rate of 204.6 events per 1000 patient-years. ECW/TBW showed the best predictive accuracy (C-index = 0.619) compared to the other indicators [*p* = 0.004, adjusted HR (95% CI) 1.70 (1.18,2.44)]. In the middle tertile (0.385–0.405), ECW/TBW had a strong independent negative association with patient survival [adjusted HR (95% CI) 2.88 (1.39–5.97),* p* = 0.004]. Patients who had a high ECW/TBW (ECW/TBW ≥ 0.395) combined with a high NLR had 3.84-fold risk of mortality (*p* < 0.001, 95% CI 1.99,7.38).

**Conclusions:**

ECW/TBW was better than other indicators in predicting survival of cancer patients with sarcopenia. High ECW/TBW combined with high NLR would further increase the risk of mortality.

*Trial registration*: The Investigation on Nutrition Status and Clinical Outcome of Common Cancers (INSCOC) (Chinese Clinical Trial Registry: ChiCTR1800020329, URL of registration: http://www.chictr.org.cn/showprojen.aspx?proj=31813).

**Supplementary Information:**

The online version contains supplementary material available at 10.1186/s12986-022-00667-3.

## Introduction

Patients with advanced cancer often experience a degenerative loss of skeletal muscle mass and strength. The Asian Working Group for Sarcopenia developed the Asian consensus for sarcopenia. Sarcopenia is defined as low muscle mass plus low muscle strength (e.g., reduced handgrip strength) or low physical performance (e.g., reduced gait speed) [1]. Patients with sarcopenia experience a poor quality of life and notable symptoms of depression [2]. Aging, inflammation, and inadequate nutrition pose a severe threat to skeletal muscle health and function [3], and sarcopenia is becoming a serious threat to older adults. A previous report showed that older people with sarcopenia are prone to multimorbidity, frailty, and an increased risk of functional impairment [4]. Sarcopenia was detected to be notably prevalent among patients with cancer [5]. Sarcopenia is independently associated with reduced overall survival in patients with cancer [6].

Bioelectrical impedance analysis (BIA) is a method that passes a weak electrical current through the body to estimate body composition and has been widely used to evaluate the nutritional state of patients [7, 8]. In breast tumors, mapping of the water fraction in each compartment of the tumor and intricate water diffusion were used to monitor changes during tumor progression and to assess tumor response to drugs [9]. The water fraction in each compartment may reveal information about the body. Extracellular water (ECW) and the ECW/total body water (TBW) ratio are significantly associated with severity of nutritional status, and ECW/TBW is an indicator for edema in patients [10, 11]. ECW/TBW was shown to be negatively associated with serum albumin and hemoglobin levels and duration of mechanical ventilation [12]. Intracellular water (ICW) reflects the body cell mass especially muscle mass [13, 14]. In healthy people, weight reduction does not change ECW or TBW, and ECW/TBW increases during weight maintenance after weight reduction [15]. A study reported that the ECW compartment and ECW/ICW are larger in healthy older subjects independent of sex, lean soft tissue, and fat mass [16]. A recent study showed that patients with low fat-free mass (FFM) have a worse prognosis and that FFM is significantly associated with nutritional status [17]. A retrospective study of patients with chronic obstructive pulmonary disease showed that patients with active cell mass (ACM) depletion had a higher death rate than those without ACM depletion [18]. Considering the influence of inflammation on cancer progression, neutrophil-to-lymphocyte ratio (NLR) may be a suitable biomarker of Inflammation [19].

Our study aimed to find out an optimal body water indicator among 8 indicators and evaluate the relationship between the indicator and the prognosis of cancer patients with sarcopenia.

## Patients and methods

### Study population and design

Patients in this study were derived from the Investigation on Nutrition Status and Clinical Outcome of Common Cancers (INSCOC)(Chinese Clinical Trial Registry: ChiCTR1800020329) project of China [20]. INSCOC is a large-scale, long-term follow-up prospective study of patients aged > 18 years with cancer. INSCOC aims to help diagnose malnutrition in patients with cancer in China and to identify the risk factors associated with negative outcomes. From 2013 to 2020 the INSCOC study recruited patients enrolled at more than 100 clinical centers throughout China. The primary outcome of interest was all-cause mortality presented as the longest time to follow-up available. The mean follow-up time of the patients included in the current study was 46 months. The INSCOC study was approved by the medical ethical review committee of the registered hospitals and was conducted in accordance with the Declaration of Helsinki.

### Patient characteristics

The current analysis included 287 cancer patients with sarcopenia who underwent body composition analysis and without fluid retention (Additional file 1: Fig. S1). We exclude all patients without tumor stage (n = 18). Baseline patient characteristics included age, sex, body mass index (BMI), tumor type, tumor stage, patient-generated subjective global assessment (PG-SGA), NLR, FFM, TBW, ECW, ICW. Tumor stage was evaluated according to the 8th edition of the American Joint Committee on Cancer TNM staging system [19].

### Diagnosis of sarcopenia

In accordance with previously published studies, sarcopenia was defined as a low appendicular skeletal muscle index (ASMI) and low handgrip strength (HGS < 28 kg for men and HGS < 18 kg for women) [1]. ASMI was defined as appendicular skeletal muscle mass (ASM) divided by height-squared. ASM was estimated using an equation previously validated in a Chinese population: ASM = 0.193 × weight (kg) + 0.107 × height (cm) − 4.157 × sex (male = 1, female = 2) − 0.037 × age (year) − 2.631. The agreement between the ASM equation model and dual X-ray absorptiometry is good (adjusted R^2^ = 0.90, standard error of estimate = 1.63 kg) [21]. The cutoff for defining low muscle mass was based on the lowest 20% percentile of the study population for each sex (ASMI < 6.946 for men and ASMI < 5.421 for women) [22].

### Assessment of anthropometric and laboratory measurements

In our study, body composition analyses, including FFM, TBW, ECW, and ICW, were conducted using an InBody S10 analyzer (Biospace Co., Ltd., Seoul, Korea). The anthropometric information, existing comorbidities, general information, nutrition related information and the medical history were collected for all patients within the first 48 h after admission by professionals. Laboratory measurements, including neutrophil, lymphocyte, and albumin levels were obtained after at least 9 h of fasting within 24 h of hospitalization. It has been shown that ACM (FFM—ECW) is a major determinant of resting energy expenditure [18].NLR was calculated using the following formula: NLR = neutrophil (× 10^9^)/lymphocyte (× 10^9^). PG-SGA was used to measure the nutritional status of the patients.

### Statistical analysis

Baseline characteristics are presented as median (interquartile range) for continuous variables and as number (proportions) for categorical variables. Data analysis was performed using R (version 4.0.2, http://www.rproject.org). Differences in baseline characteristics between men and women were compared using the χ^2^ test for categorical variables and the two-sample Wilcoxon rank-sum test for continuous variables because the continuous data are all in a skew distribution. The discriminatory ability and prediction accuracy of each factor were assessed using the C-index and time-dependent receiver operating characteristic (ROC) curves. A calibration curve was prepared to verify the ECW/TBW results. Nonlinear effects were modeled using a restricted quartic spline. The tertile of ECW/TBW was based on the threshold of the restricted quartic spline. The cutoff value of ECW/TBW was based on maximally selected rank statistics using the “survminer” package. The cutoff value of NLR was based on previous studies. Hazard ratios (HRs) and 95% confidence intervals (CIs) of patients were estimated by modeling risk factors as continuous variables and modeling ECW/TBW to per SD (standard deviation) using univariate Cox regression models or multivariate Cox regression models, with and without adjustment for matched variables (age, sex, tumor stage, tumor type, BMI, PG-SGA, and NLR). Adjusted variables were selected based on clinical experience and published studies. Sensitivity analysis excluded patients with kidney disease to exclude associated disease interference, and another sensitivity analysis excluded patients survival less than 3 month to exclude the interference caused by metabolic disorders in patients with end-stage cancer. Sensitivity analyses were performed to assess the stability of the results. Heterogeneity in subgroups was assessed by simultaneous multivariate Cox regression and is presented in forest plots; interactions between subgroups and ECW/TBW were examined by likelihood ratio testing to demonstrate the relationship between body composition and other cancer-related factors. Kaplan–Meier curves and were multivariate Cox regression used to perform survival analysis of the combined analysis of ECW/TBW and NLR.

For practical reasons, an increase in the AUC of 0.025 per additional risk factor is considered clinically relevant [23]. A 2-tailed *p* < 0.05, was considered to be statistically significant in all analyses.

## Results

### Characteristics of patients

The median (interquartile range) age of patients was 65 [12]. Among the 287 patients, 141 were digestive system cancer (51 colorectal, 49 gastric, 19 esophageal, and 22 other digestive system cancer), 90 were lung cancer, and 56 were other cancer. 35 were in tumor stage I, 74 were in tumor stage II, 73 were in tumor stage III, 105 were in tumor stage IV. 140 deaths were observed during the follow-up period. 158 patients received surgical treatment, 173 patients received chemotherapy and 30 patients received radiotherapy. The male patients (138, 48.1%) in the study population tended to have a higher NLR [3.59 (3.96) vs 2.69 (3.66); *p* = 0.027], FFM [42.50 (5.80) vs 32.70 (4.40); *p* < 0.001], TBW [33.55 (4.70) vs 25.60 (3.00); *p* < 0.001], ECW [13.10 (1.97) vs 10.20 (1.40); *p* < 0.001], ICW [20.40 (2.77) vs 15.60 (2.20); *p* < 0.001], and ACM [29.80 (4.45) vs 22.70 (3.20); *p* < 0.001] than female patients (149, 51.9%). However, there were no significant differences between men and women in ECW/TBW, ICW/TBW or ECW/ICW (Additional file 1: Table S1).

### Discriminatory ability and prediction accuracy of body water composition of cancer patients with sarcopenia

**Fig. 1:**
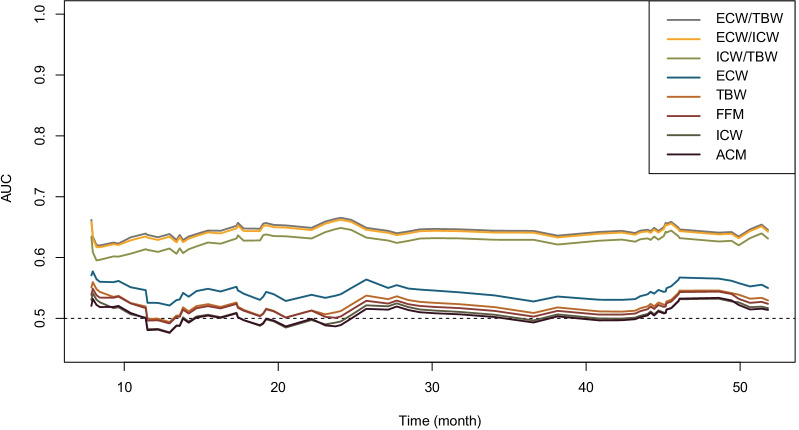
Time-dependent AUC values of different factors regarding body water. *Notes*: AUC: area under the curve, ECW/TBW: extracellular water/total body water, ECW/ICW: extracellular water/intracellular water, ICW/TBW: intracellular water/total body water, ECW: extracellular water, TBW: total body water, FFM: free fat mass, ICW: intracellular water, ACM: active cell mass

The C-index (95%CI) of ECW/TBW, ECW/ICW, ICW/TBW, ECW, TBW, FFM, ICW, and ACM were 0.619 (0.568–0.670), 0.617 (0.566–0.668), 0.605 (0.553–0.656), 0.533 (0.484–0.582), 0.517 (0.468–0.566), 0.512 (0.463–0.560), 0.506 (0.457–0.555), and 0.502 (0.453–0.550), respectively (Additional file 1: Table S2). Figure 1 shows that ECW/TBW had the best prediction accuracy based on the AUC in time-dependent ROC analysis. Additional file 1: Fig. S2 shows a good agreement between the predicted and observed outcomes of ECW/TBW. Combined with ECW/TBW, the prediction accuracy of tumor stage greatly improved (1-year AUC from 0.680 to 0.714; 2-year AUC from 0.735 to 0.769; 3-year AUC from 0.777 to 0.801, and 4-year AUC from 0.783 to 0.801) (Additional file 1: Fig. S3).

### Association between ECW/TBW and the survival of cancer patients with sarcopenia

**Fig. 2:**
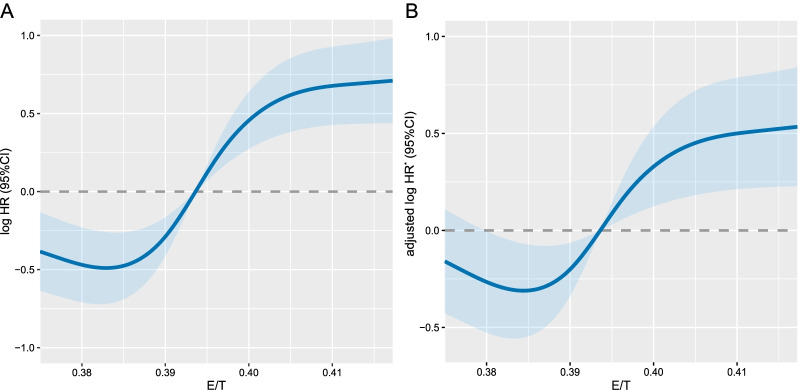
The relation of ECW/TBW and survival of cancer patients with sarcopenia. *Notes*: HRs of survival of cancer patients with sarcopenia relation to ECW/TBW (as continue value) were calculated using univariate Cox regression model (**A**) or multivariate Cox regression model (**B**). Each subgroup analysis adjusted for age, sex, tumor stage, tumor type, BMI, PG-SGA, and NLR if not stratified by these variables. ECW/TBW: extracellular water/total body water, PG-SGA: patient-generated subjective global assessment, NLR: neutrophil-to-lymphocyte ratio

There was a negative association between ECW/TBW (cutoff value = 0.395) and patient survival [adjusted HR (95% CI) 1.70 (1.18–2.44), *p* = 0.004]. The different characteristics of patients with either low ECW/TBW or high ECW/TBW are shown in Additional file 1: Table S3. Patients in the fourth quartile of ECW/TBW had a higher risk of mortality [Q4 (ECW/TBW ≥ 0.400): adjusted HR (95% CI) 1.49 (1.01–2.19), *p* = 0.043] (Table 1, Fig. 2). Two sensitivity analyses were performed to assess this outcome. The sensitivity analyses excluded the patients with a survival of less than 3 months [by cutoff value: adjusted HR (95% CI) 1.70 (1.18–2.44),* p* = 0.025; Q4: adjusted HR (95% CI) 1.74 (0.90–3.37), *p* for trend = 0.041] and excluded patients with chronic kidney disease, edema, or ascites [by cutoff value: adjusted HR (95% CI) 1.63 (1.11–2.42),* p* = 0.014; Q4: adjusted HR (95% CI) 1.70 (0.93–3.09), *p* for trend = 0.027] (Additional file 1: Table S4).

**Table 1:** Univariate and multivariate analysis on the overall survival of ECW/TBW in patients with cancer sarcopenia

Characteristics	Cases	Crude HR (95%CI)	*p* value	Adjusted HR (95%CI)	*p* value
Per SD	287	1.30 (1.07–1.58)	0.008	1.13 (0.89–1.42)	0.310
By cut-off value					
< 0.395	170		Ref		Ref
≥ 0.395	117	2.25 (1.61–3.14)	< 0.001	1.70 (1.18–2.44)	0.004
By quartile					
Q1–Q3 (< 0.400)	221		Ref		Ref
Q4 (≥ 0.400)	66	1.93 (1.34–2.76)	< 0.001	1.49 (1.01–2.19)	0.043

Hazard ratios (HRs) of survival for cancer patients with sarcopenia were calculated using univariate Cox regression model or multivariate Cox regression model. Each subgroup analysis included ECW/TBW (as a continuous variable) as the independent variable and adjusted for age, sex, tumor stage, tumor type, BMI, PG-SGA, and NLR if not stratified by these variables. ECW/TBW: extracellular water/total body water, PG-SGA: patient-generated subjective global assessment, NLR: neutrophil-to-lymphocyte ratio

### Threshold analysis between ECW/TBW and the survival of cancer patients with sarcopenia

**Fig. 3:**
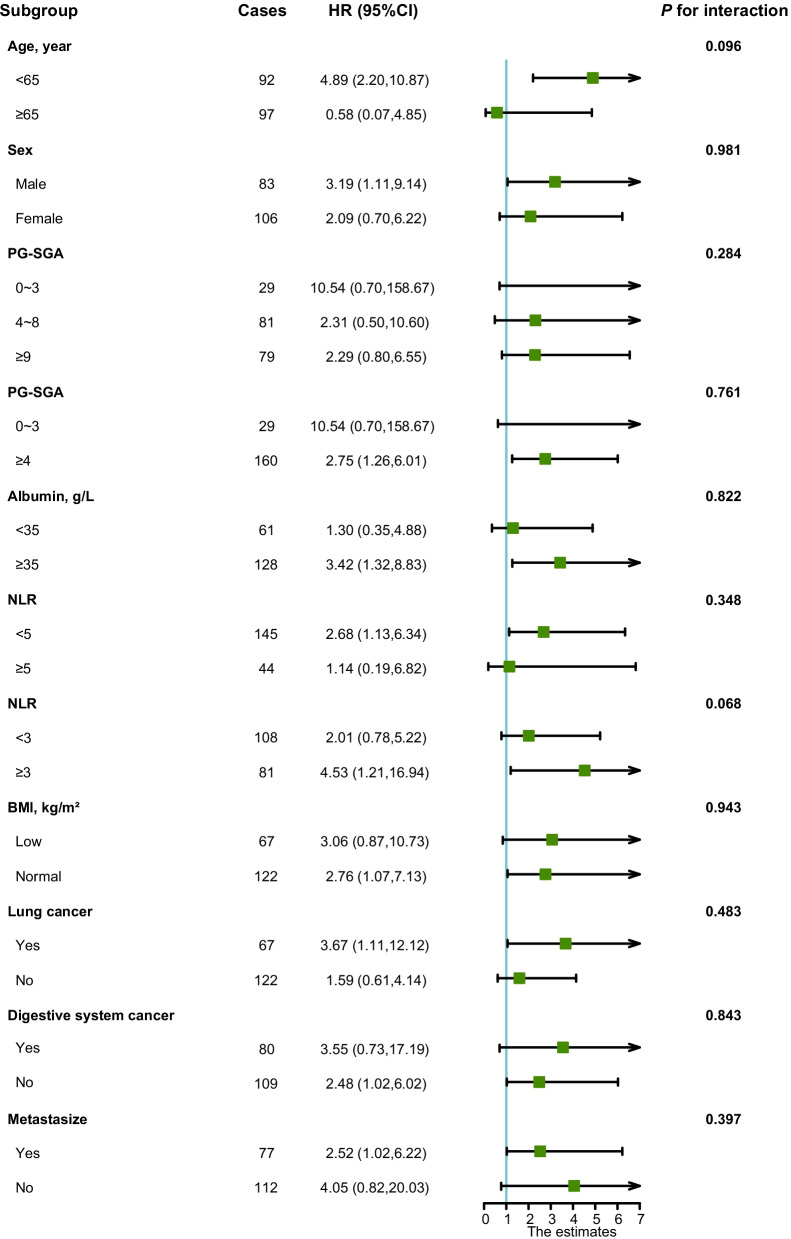
The association between ECW/TBW (0.385–0.405) and the risk of survival of cancer patients with sarcopenia in various subgroups. *Notes*: HRs of survival of cancer patients with sarcopenia relation to ECW/TBW (as continue value) were calculated using multivariate Cox regression models. Each subgroup analysis adjusted for age, sex, tumor stage, tumor type, BMI, PG-SGA, and NLR. ECW/TBW: extracellular water/total body water, PG-SGA: patient-generated subjective global assessment, NLR: neutrophil-to-lymphocyte ratio

Based on the spline plots, the ECW/TBW tertiles were 0.385 and 0.405, respectively. The threshold analysis showed that in the first tertile, ECW/TBW had a positive association with patient survival [adjusted HR (95% CI) 0.39 (0.19–0.79),* p* = 0.009]; in the middle tertile, ECW/TBW had a strong negative association with patient survival [adjusted HR (95% CI) 2.88 (1.39–5.97),* p* = 0.004], and in the last tertile, the ECW/TBW was not associated with the survival of cancer patients with sarcopenia [adjusted HR (95% CI) 1.23 (0.85–1.80),* p* = 0.277] (Table 2). The same trend was observed in the different subgroups (Fig. 3, Additional file 1: Table S5).

**Table 2:** Univariate and multivariate analysis on the overall survival of ECW/TBW tertiles in patients with cancer sarcopenia

Characteristics	Cases	Crude HR (95%CI)	*p* value	Adjusted HR (95%CI)	*p* value
< 0.385	50	0.60 (0.40–0.90)	0.014	0.39 (0.19–0.79)	0.009
0.385 ~ 0.405	189	3.42 (1.71–6.82)	< 0.001	2.88 (1.39–5.97)	0.004
≥ 0.405	48	1.27 (0.90–1.79)	0.180	1.23 (0.85–1.80)	0.277

Hazard ratios (HRs) of survival of cancer patients with sarcopenia were calculated using univariate Cox regression model or multivariate Cox regression model. Each subgroup analysis included ECW/TBW (as a continuous variable) and adjusted for age, sex, tumor stage, tumor type, BMI, PG-SGA, and NLR if not stratified by these variables. ECW/TBW: extracellular water/total body water, PG-SGA: patient-generated subjective global assessment, NLR: neutrophil-to-lymphocyte ratio

### Association between the combination of ECW/TBW and NLR and the survival of cancer patients with sarcopenia

Compared with patients with low ECW/TBW and low NLR, patients with high ECW/TBW and high NLR had the highest risk of death [adjusted HR (95% CI) 3.84 (1.99–7.38), *p* < 0.001], and the comparison between the four groups is shown in the Kaplan–Meier curve (log-rank *p* < 0.001) (Additional file 1: Fig. S4).

### Association between the combination of ECW/TBW and PG-SGA and the survival of cancer patients with sarcopenia

Compared with patients with low ECW/TBW and well nourished, patients with high ECW/TBW and malnutrition had the highest risk of death, and the comparison between the four groups is shown in the Kaplan–Meier curve (log-rank *p* < 0.001) (Additional file 1: Fig. S5).

## Discussion

Cancer patients with sarcopenia experience a rapid increase in mortality if they exceeded this threshold of ECW/TBW (ECW/TBW ≥ 0.385). Previous studies have shown that body water could provide useful information and is associated with mortality [24–26]. Some investigators found ICW was associated with body cell mass, especially muscle mass, and was independent of muscle mass [13, 14, 27, 28]. FFM and ECW/TBW have been revealed to be associated with malnutrition [17, 29]. ECW/TBW was also shown to indicate edema in patients [12]. Among the eight characteristics of body composition, ECW/TBW had the best C-index and the best AUC of time-dependent ROC in the follow-up period. The threshold for ECW/TBW was 0.385 in the cancer patients with sarcopenia. Sensitivity analysis showed the same results.

ECW/TBW was shown to be independently associated with coronary artery calcification in patients with chronic kidney disease [30], and ECW/TBW could predict the body fluid response to the SGLT2 inhibitor dapagliflozin and assess all-cause mortality, nutrition status and body composition in patients with diabetic kidney disease [31]. ECW/TBW was shown to be a predictor of relative dose intensity in patients with hepatocellular carcinoma treated with lenvatinib [32]. During fluid resuscitation, ECW/TBW and ICW/TBW showed dehydration and edema in non-surviving patients with sepsis [33]. In type 2 diabetes, a higher ECW/TBW was associated with poorer cognitive function [34]. Increased in ECW/TBW has been associated with progressive liver fibrosis and malnutrition and is related to the prognosis of cirrhotic patients [35]. In the hemodialysis population, ECW/TBW reference values from 0.390 to 0.410 are the most promising (based on InBody S10) [10]. ECW/TBW can be useful for the long-term maintenance of lymphedema, and the cutoff values of ECW/TBW for moderate and severe degree cancer treatment-related lymphedema were 0.3855 and 0.3955, respectively (based on InBody S10) [36]. ECW/TBW may be an objective parameter for predicting therapeutic durability in advanced lung cancer [37].

Considering that the body water is associated with BMI, age, and sex [3], the volume of the body water compartment could be notably different in different patients. The characteristics showed that distribution of body water could serve as a better prognostic factor. In the multivariate analysis, we had considered the influence of these factors, and the results indicated ECW/TBW was a significant independent risk factor for mortality. The threshold analysis showed that when ECW/TBW was less than 0.385, an increase in ECW/TBW was an independent protective factor, but when ECW/TBW was more than 0.385, an increase in ECW/TBW was an independent risk factor for non-survival. In a study, ECW/TBW showed the hydration status of patients [38], and severely overhydrated patients were defined as those with ECW/TBW > 0.400 in other studies [39, 40]. The change in ECW/TBW is very small because of homeostasis, and a small change in ECW/TBW could affect the whole body. Cancer patients with sarcopenia experience the consumption of skeletal muscle mass [41], and the threshold for those patients should be defined as 0.385, which is same as that for cancer patients with moderate lymphedema. Based on research with the same measurements, we believe that patients with cancer should maintain a lower ECW/TBW than other patients, which might be due to the protective effect of muscle and adipose mass. Malczyk, Dzięgielewska-Gęsiak [11] reported that only ECW/TBW increased significantly with age and sex in healthy older persons, especially after 65 years. Sarcopenia as an age-related process in older people involved the accelerated loss of muscle mass and function [41]. In our research, in our study, the ECW/TBW of cancer patients with sarcopenia should be maintained at a low level. This result indicates that cancer patients with sarcopenia need higher levels of nutrition, especially muscle mass. In the subgroup of age ≥ 65, ECW/TBW showed a stronger trend of protective effect, which was consistent with this results.

For patients with cancer, inflammatory and nutritional states are important prognostic factors that affect survival [42–46]. In previous studies, it was found that NLR, as the easiest and cheapest inflammatory parameters to obtain, has a good ability to indicate the patient's systemic inflammation status, and sarcopenia combined with inflammation nearly doubled risk of death [47, 48]. Considering that inflammation status is a significant prognostic factor for cancer patients [43], we found that the combination of ECW/TBW and NLR is effective for predicting survival. Higher levels of inflammation will affect the muscle mass and prognosis of cancer patients. Higher levels of inflammation with higher ECW/TBW may indicate that patients have a great nutritional risk and a worse prognosis. Researchers had found that the malnutrition was associated with greater ratio of ECW [49, 50]. We found higher ECW/TBW showed worse prognosis in patients with malnutrition.

The present study has several limitations. First, the small sample size from a hospital based study may have been a source of bias, including selection bias and recall bias. Second, we were unable to evaluate the patients’ body composition using the dual X-ray absorptiometry, and the findings of this study can only be generalized to the same measurements. The volume of body water was differed with different measurements, but the ratio of ECW to TBW might be similar because the distribution of body water could exclude the differences in measurement. Further prospective studies are needed to confirm whether the dynamic change in ECW/TBW could be a better prediction tool, and whether ECW/TBW could be similar to the different measurements.

In conclusion, the prognostic value of ECW/TBW was better than TBW, ECW, ICW, FFM, ACM, ECW/ICW, and ICW/TBW in cancer patients with sarcopenia. The ECW/TBW was an independent prognostic factor for OS in cancer patients with sarcopenia and high level of ECW/TBW was significantly associated with the mortality of cancer patients with sarcopenia. Cancer patients with sarcopenia experience a rapid increase in mortality if they exceeded this threshold of ECW/TBW (ECW/TBW ≥ 0.385). We also found that patients with combined high ECW/TBW and high NLR have the worst prognosis.

## Supplementary Information

**Additional file 1**. Supplemenrtary Tables and Figures.

## Data Availability

Data described in the manuscript, code book, and analytic code will be made available upon request pending application and approval.

## Abbreviations

ACM: Active cell mass
ASM: Appendicular skeletal muscle mass
ASMI: Appendicular skeletal muscle index
AUC: Area under the curve
BIA: Bioelectrical impedance analysis
BMI: Body mass index
CI: Confidence intervals
ECW: Extracellular water
ECW/ICW: Extracellular water/intracellular water
ECW/TBW: Extracellular water/total body water
FFM: Free fat mass
HGS: Handgrip strength
HR: Hazard ratio
ICW: Intracellular water
ICW/TBW: Intracellular water/total body water
INSCOC: The Investigation on Nutrition Status and Clinical Outcome of Common Cancers
NLR: Neutrophil–lymphocyte ratio
PG-SGA: Patient-generated subjective global assessment
ROC: Receiver operating characteristic
TBW: Total body water
