# Supplementary material for: Extracellular water to total body water ratio predicts survival in cancer patients with sarcopenia: a multi-center cohort study
Source: Nutr Metab (Lond). 2022 May 7;19:34. doi: 10.1186/s12986-022-00667-3 (PMC9077863; doi:10.1186/s12986-022-00667-3)
Supplement: Supplementary file 1 — Additional file 1. Supplemenrtary Tables and Figures. [file 12986_2022_667_MOESM1_ESM.docx]

**

**

**Figure S1.** Flow chart.


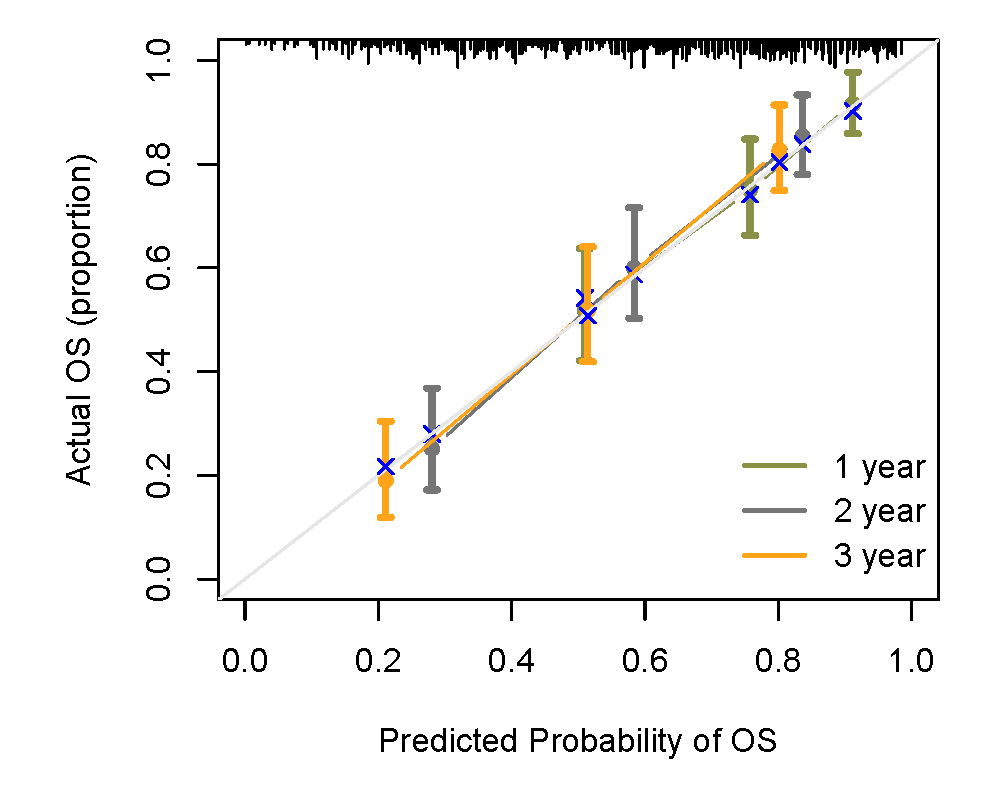


**Figure S2.** Calibration plot for ECW/TBW in different times of survival.

Notes: OS: overall survival.


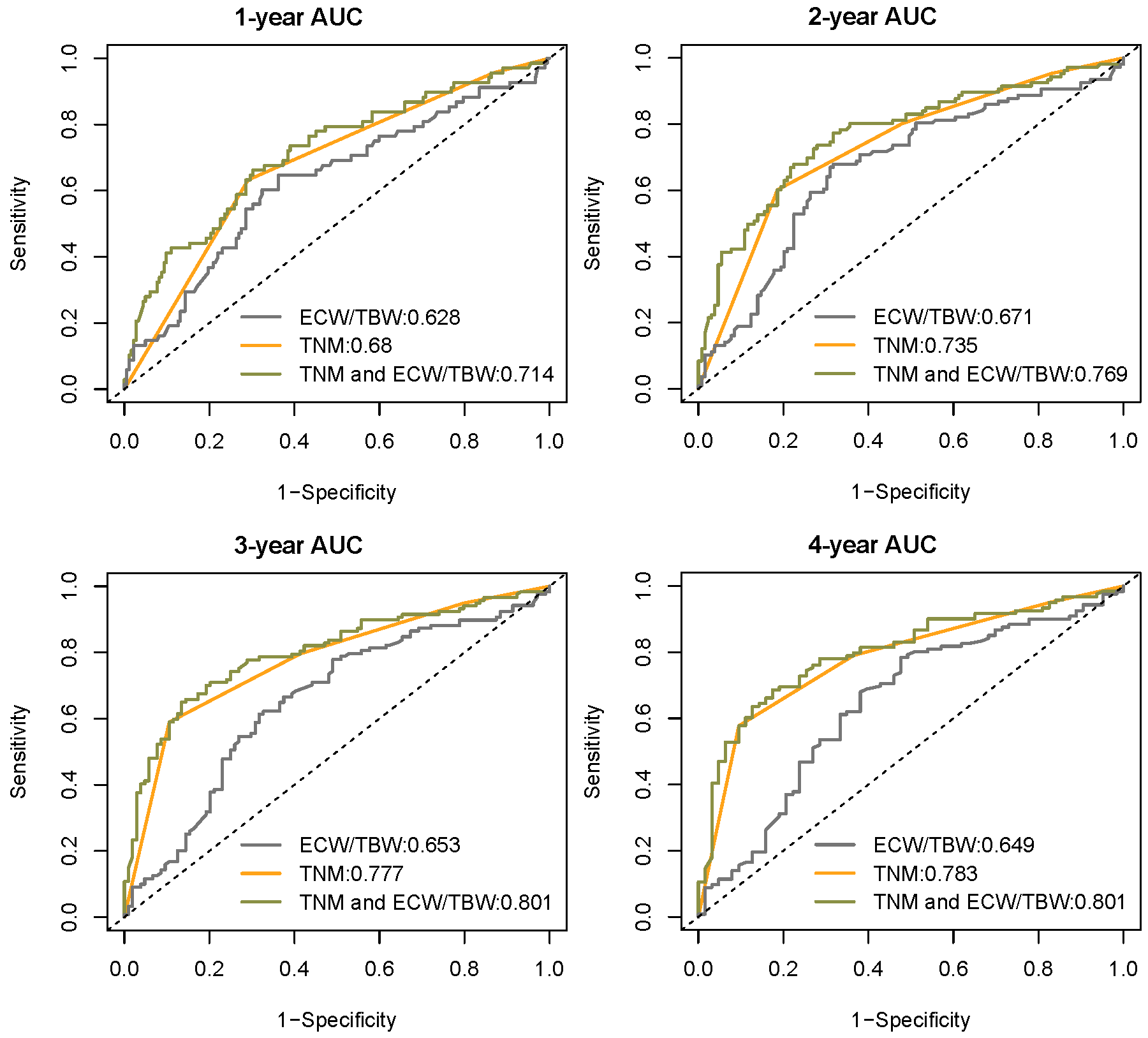


**Figure S3.** Comparing improvements in the TNM stage in predicting prognosis.

Notes: AUC: area under the curve.


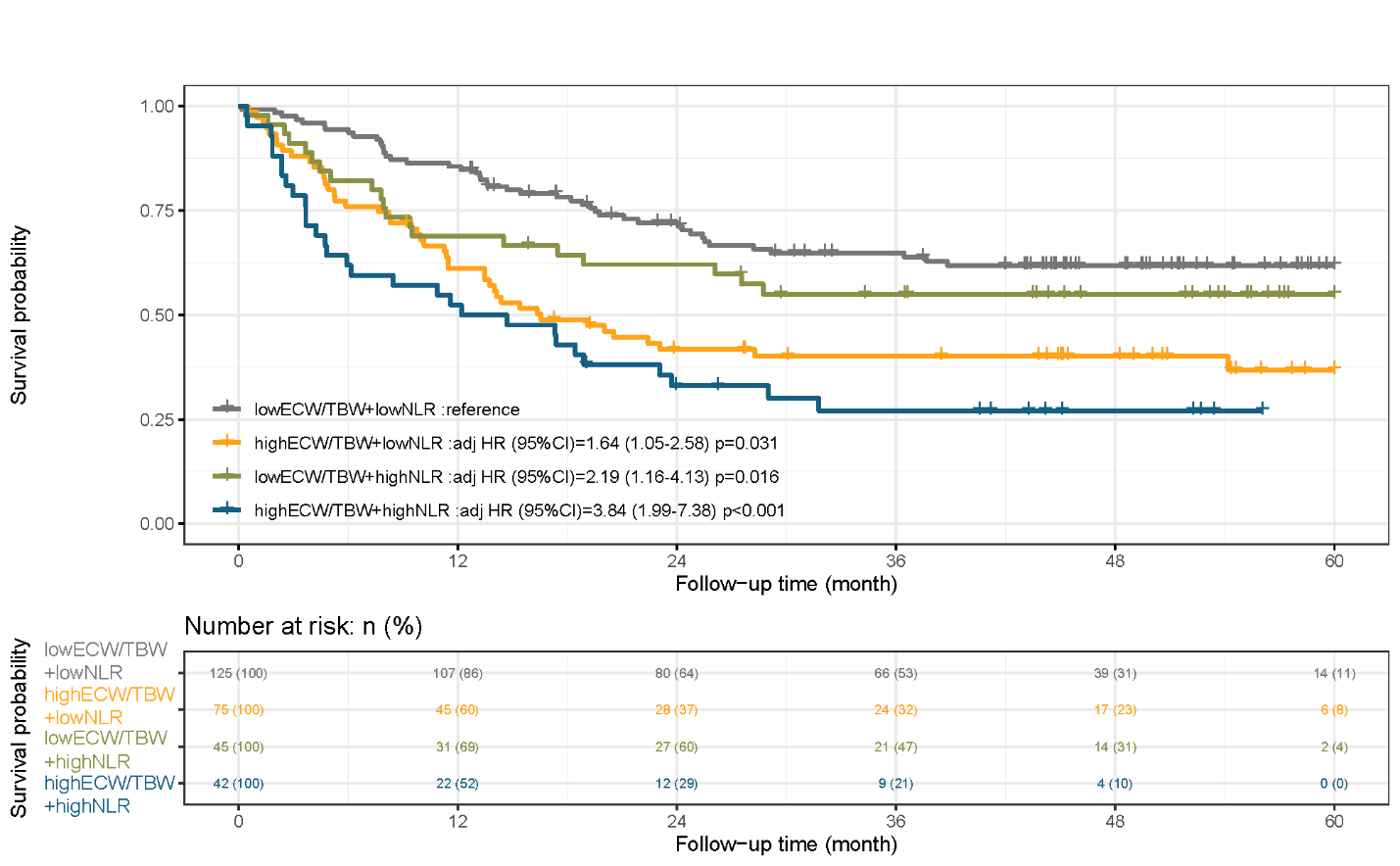


**Figure S4.** Results of the Kaplan-Meier survival analysis for cancer patients with sarcopenia stratified by ECW/TBW (cut-off value) and NLR (NLR=5).

Notes: HRs of survival of cancer patients with sarcopenia relation to ECW/TBW (as continue value) were calculated using multivariate Cox regression models. Each subgroup analysis adjusted for age, sex, tumor stage, tumor type, BMI, PG-SGA, and NLR. ECW/TBW: extracellular water/ total body water, PG-SGA: patient-generated subjective global assessment, NLR: neutrophil-to-lymphocyte ratio.


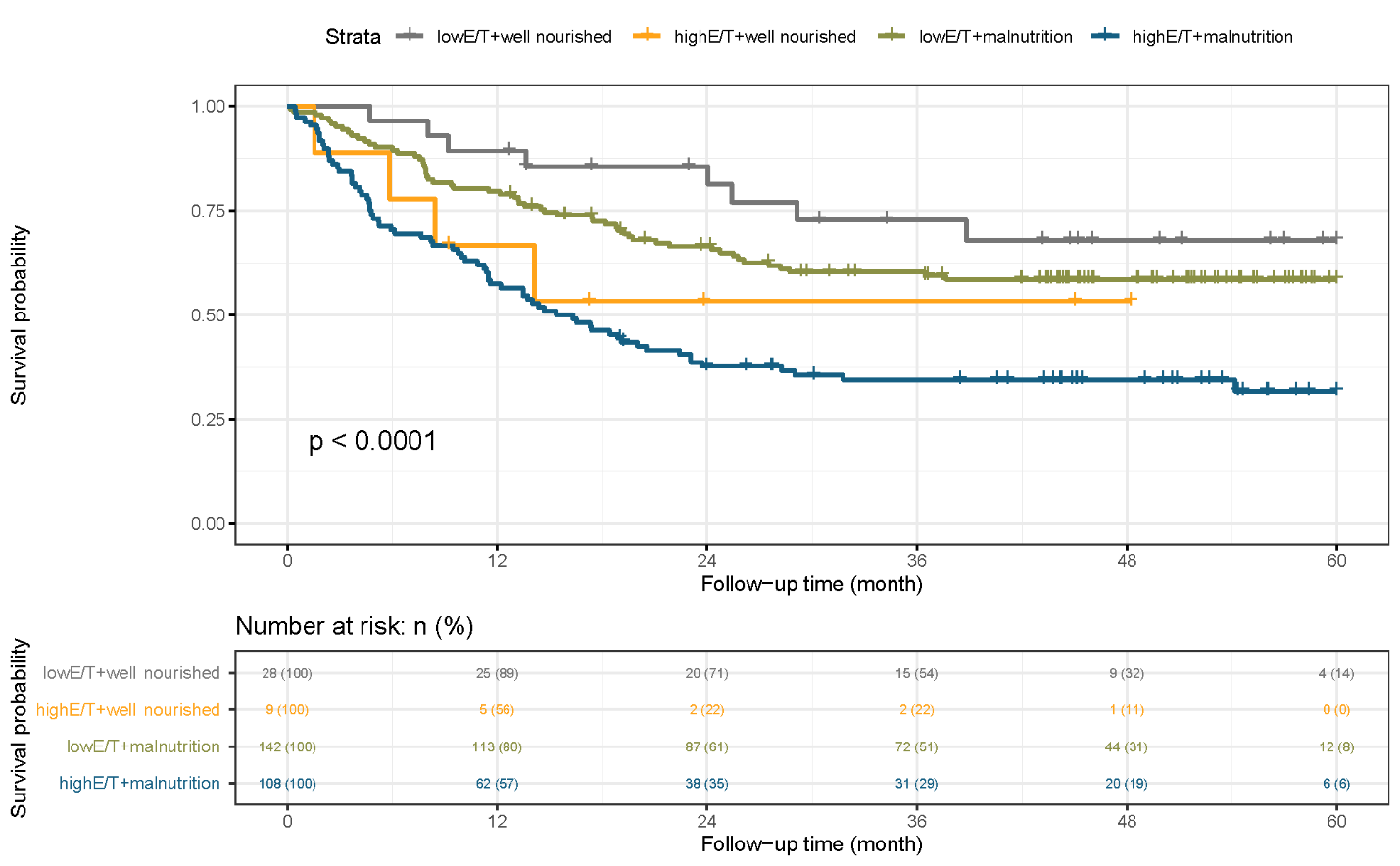


**Figure S5.** Results of the Kaplan-Meier survival analysis for cancer patients with sarcopenia stratified by ECW/TBW (cut-off value) and malnutrition.

Notes: HRs of survival of cancer patients with sarcopenia relation to ECW/TBW (as continue value) were calculated using multivariate Cox regression models. Each subgroup analysis adjusted for age, sex, tumor stage, tumor type, BMI, PG-SGA, and NLR. ECW/TBW: extracellular water/ total body water, PG-SGA: patient-generated subjective global assessment, NLR: neutrophil-to-lymphocyte ratio.

**Table S1.** Baseline characteristics of the study population.

| **Characteristics** | **Overall** | **Male** | **Female** | ***p* value** |
| --- | --- | --- | --- | --- |
|  | **n=287** | **n=138** | **n=149** |  |
| Age, year | 65.00 [12.00] | 66.00 [12.75] | 64.00 [13.00] | 0.020 |
| BMI, kg/m^2^ | 18.84 [2.15] | 18.78 [1.51] | 18.94 [2.63] | 0.098 |
| Tumor type | |  |  | <0.001 |
| Lung cancer | 90 (31.4) | 52 (37.7) | 38 (25.5) |  |
| Digestive system cancer | 141 (49.1) | 82 (59.4) | 59 (39.6) |  |
| Other cancers | 56 (19.5) | 4 (2.9) | 52 (34.9) |  |
| Tumor stage | |  |  | 0.097 |
| I | 35 (12.2) | 10 (7.2) | 25 (16.8) |  |
| II | 74 (25.8) | 36 (26.1) | 38 (25.5) |  |
| III | 73 (25.4) | 37 (26.8) | 36 (24.2) |  |
| IV | 105 (36.6) | 55 (39.9) | 50 (33.6) |  |
| PG-SGA |  |  |  | 0.040 |
| 0~3 | 37 (12.9) | 14 (10.1) | 23 (15.4) |  |
| 4~8 | 120 (41.8) | 51 (37.0) | 69 (46.3) |  |
| ≥9 | 130 (45.3) | 73 (52.9) | 57 (38.3) |  |
| Surgery |  |  |  | 0.557 |
| No | 129 (44.9) | 65 (47.1) | 64 (43.0) |  |
| Yes | 158 (55.1) | 73 (52.9) | 85 (57.0) |  |
| Chemotherapy | |  |  | 0.374 |
| No | 114 (39.7) | 59 (42.8) | 55 (36.9) |  |
| Yes | 173 (60.3) | 79 (57.2) | 94 (63.1) |  |
| Radiotherapy | |  |  | 0.977 |
| No | 257 (89.5) | 123 (89.1) | 134 (89.9) |  |
| Yes | 30 (10.5) | 15 (10.9) | 15 (10.1) |  |
| NLR | 3.08 [3.95] | 3.59 [3.96] | 2.69 [3.66] | 0.027 |
| FFM | 36.10 [10.25] | 42.50 [5.80] | 32.70 [4.40] | <0.001 |
| TBW | 28.20 [7.95] | 33.55 [4.70] | 25.60 [3.00] | <0.001 |
| ECW | 11.10 [3.05] | 13.10 [1.97] | 10.20 [1.40] | <0.001 |
| ICW | 17.10 [5.10] | 20.40 [2.77] | 15.60 [2.20] | <0.001 |
| ACM | 25.00 [7.35] | 29.80 [4.45] | 22.70 [3.20] | <0.001 |
| ICW/TBW | 0.61 [0.01] | 0.61 [0.01] | 0.61 [0.01] | 0.563 |
| ECW/TBW | 0.39 [0.01] | 0.39 [0.01] | 0.39 [0.01] | 0.797 |
| ECW/ICW | 0.65 [0.04] | 0.65 [0.04] | 0.65 [0.04] | 0.710 |

For continuous variables, values are median [interquartile range] for categorical variables, values are expressed as n (%). Differences in baseline characteristics were compared using χ2 test for categorical variables and t Wilcoxon ranked sum test for continuous variables. PG-SGA: patient-generated subjective global assessment, NLR: neutrophil-to-lymphocyte ratio. FFM: free fat mass, TBW: total body water, ECW: extracellular water, ICW: intracellular water, ACM: active cell mass, ICW/TBW: intracellular water/ total body water, ECW/TBW: extracellular water/ total body water, ECW/ICW: extracellular water/ intracellular water.

**Table S2.** C-index of different factors regarding body water.

| **Characteristics** | ***C*-index (****95%CI)** |
| --- | --- |
| ECW/TBW | 0.619 (0.568-0.670) |
| ECW/ICW | 0.617 (0.566-0.668) |
| ICW/TBW | 0.605 (0.553-0.656) |
| ECW | 0.533 (0.484-0.582) |
| TBW | 0.517 (0.468-0.566) |
| FFM | 0.512 (0.463-0.560) |
| ICW | 0.506 (0.457-0.555) |
| ACM | 0.502 (0.453-0.550) |

ECW/TBW: extracellular water/ total body water, ECW/ICW: extracellular water/ intracellular water, ICW/TBW: intracellular water/ total body water, ECW: extracellular water, TBW: total body water, FFM: free fat mass, ICW: intracellular water, ACM: active cell mass.

**Supplementary Table 3.** Detailed characteristics of the low or high E/T population.

| **Characteristics** | **low E/T** | **high E/T** | ***p* value** |
| --- | --- | --- | --- |
|  | **n=170** | **n=117** |  |
| Gender |  |  | 0.954 |
| Male | 81 (47.6) | 57 (48.7) |  |
| Female | 89 (52.4) | 60 (51.3) |  |
| Age, year | 62.00 [13.00] | 68.00 [12.00] | <0.001 |
| BMI, kg/m^2^ | 18.90 [1.95] | 18.77 [2.43] | 0.13 |
| Tumor type |  |  | 0.179 |
| Lung cancer | 49 (28.8) | 41 (35.0) |  |
| Digestive system cancer | 82 (48.2) | 59 (50.4) |  |
| Other cancers | 39 (22.9) | 17 (14.5) |  |
| Tumor stage |  |  | 0.145 |
| 1 | 23 (13.5) | 12 (10.3) |  |
| 2 | 50 (29.4) | 24 (20.5) |  |
| 3 | 43 (25.3) | 30 (25.6) |  |
| 4 | 54 (31.8) | 51 (43.6) |  |
| PG-SGA |  |  | 0.071 |
| 0~3 | 28 (16.5) | 9 (7.7) |  |
| 4~8 | 71 (41.8) | 49 (41.9) |  |
| >8 | 71 (41.8) | 59 (50.4) |  |
| NLR | 2.83 [3.60] | 3.54 [5.06] | 0.032 |

**Table S4.** Sensitivity analysis of ECW/TBW affecting disease-free survival and more than 3-month survival patients.

| **Exclude diseases** | **Cases** | **crude HR (95%CI)** | ***p* value** | **adjusted HR (95%CI)** | ***p* value** |
| --- | --- | --- | --- | --- | --- |
| per SD |  | 1.32 (1.07-1.63) | 0.009 | 1.11 (0.85-1.44) | 0.436 |
| by cut-off value |  |  |  |  |  |
| <0.395 | 156 |  | ref. |  | ref. |
| ≥0.395 | 95 | 2.19 (1.53-3.13) | <0.001 | 1.63 (1.11-2.42) | 0.014 |
| by quartile |  |  |  |  |  |
| Q1 (<0.387) | 67 |  | ref. |  | ref. |
| Q2 (0.387~0.394) | 65 | 1.24 (0.68-2.26) | 0.483 | 1.00 (0.54-1.87) | 0.999 |
| Q3 (0.394~0.400) | 67 | 2.47 (1.43-4.26) | 0.001 | 1.62 (0.91-2.89) | 0.102 |
| Q4 (≥0.400） | 52 | 2.71 (1.58-4.64) | <0.001 | 1.70 (0.93-3.09) | 0.083 |
| *p* for trend |  |  | <0.001 |  | 0.027 |
| **>3month** | **Cases** | **crude HR (95%CI)** | ***p* value** | **adjusted HR (95%CI)** | ***p* value** |
| per SD |  | 1.60 (1.23-2.08) | <0.001 | 1.35 (0.99-1.85) | 0.061 |
| by cut-off value |  |  |  |  |  |
| <0.395 | 163 |  | ref. |  | ref. |
| ≥0.395 | 99 | 2.08 (1.41-3.07) | <0.001 | 1.63 (1.06-2.49) | 0.025 |
| by quartile |  |  |  |  |  |
| Q1 (<0.387) | 68 |  | ref. |  | ref. |
| Q2 (0.387~0.394) | 71 | 1.48 (0.76-2.85) | 0.246 | 1.17 (0.59-2.32) | 0.663 |
| Q3 (0.394~0.400) | 69 | 2.69 (1.47-4.92) | 0.001 | 1.84 (0.98-3.45) | 0.057 |
| Q4 (≥0.400） | 54 | 2.76 (1.52-5.02) | 0.001 | 1.74 (0.90-3.37) | 0.100 |
| *p* for trend |  |  | <0.001 |  | 0.041 |

Excluded diseases/conditions included kidney disease, edema, and ascites. HRs of survival of cancer patients with sarcopenia were calculated using univariate Cox regression model or multivariate Cox regression model. Each subgroup analysis adjusted for age, sex, tumor stage, tumor type, BMI, PG-SGA, and NLR if not stratified by these variables. ECW/TBW: extracellular water/ total body water, PG-SGA: patient-generated subjective global assessment, NLR: neutrophil-to-lymphocyte ratio.

**Table S5.** Tertiles of patients’ E/T and the survival of patients with cancer sarcopenia

| **Subgroup** | **E/T < 0.385** | | **E/T = (0.385-0.405)** | | **E/T ≥ 0.405** | | ***P* for interaction** |
| --- | --- | --- | --- | --- | --- | --- | --- |
|  | **Cases** | **HR (95%CI)** | **Cases** | **HR (95%CI)** | **Cases** | **HR (95%CI)** |  |
| Age, year |  |  |  |  |  |  | 0.002 |
| <65 | 34 | 0.25 (0.05,1.14) | 92 | 4.89 (2.20,10.87) | 17 | 0.99 (0.58,1.69) |  |
| ≥65 | 16 | 0.29 (0.07,1.11) | 97 | 0.58 (0.07,4.85) | 31 | 1.71 (0.66,4.46) |  |
| Sex |  |  |  |  |  |  | 0.033 |
| Male | 29 | 0.49 (0.22,1.06) | 83 | 3.19 (1.11,9.14) | 26 | 1.16 (0.73,1.85) |  |
| Female | 21 | >1000 (0.00,Inf) | 106 | 2.09 (0.70,6.22) | 22 | 1.58 (0.37,6.69) |  |
| PGSGA |  |  |  |  |  |  | 0.172 |
| 0~3 | 7 | 0.15 (0.00,Inf) | 29 | 10.54 (0.70,158.67) | 1 | NA (NA,NA) |  |
| 4~8 | 19 | 0.00 (0.00,Inf) | 81 | 2.31 (0.50,10.6) | 20 | 0.77 (0.37,1.59) |  |
| >8 | 24 | 0.32 (0.11,0.94) | 79 | 2.29 (0.80,6.55) | 27 | 1.43 (0.63,3.26) |  |
| PGSGA |  |  |  |  |  |  | 0.998 |
| 0~3 | 7 | 0.15 (0.00,Inf) | 29 | 10.54 (0.70,158.67) | 1 | NA (NA,NA) |  |
| ≥4 | 43 | 0.38 (0.17,0.87) | 160 | 2.75 (1.26,6.01) | 47 | 1.19 (0.78,1.80) |  |
| Albumin, g/L | |  |  |  |  |  | 0.771 |
| <35 | 18 | 0.00 (0.00,Inf) | 61 | 1.30 (0.35,4.88) | 31 | 2.16 (0.71,6.58) |  |
| ≥35 | 32 | 0.48 (0.24,0.95) | 128 | 3.42 (1.32,8.83) | 17 | 1.75 (0.69,4.42) |  |
| NLR |  |  |  |  |  |  | 0.866 |
| <5 | 34 | 0.19 (0.04,0.89) | 145 | 2.68 (1.13,6.34) | 21 | 1.23 (0.40,3.81) |  |
| ≥5 | 16 | 1.25 (0.27,5.79) | 44 | 1.14 (0.19,6.82) | 27 | 1.31 (0.82,2.10) |  |
| NLR |  |  |  |  |  |  | 0.344 |
| <3 | 19 | 2.08 (0.00,67.03) | 108 | 2.01 (0.78,5.22) | 13 | 1.07 (0.21,5.49) |  |
| ≥3 | 31 | 0.84 (0.31,2.25) | 81 | 4.53 (1.21,16.94) | 35 | 1.31 (0.84,2.05) |  |
| BMI, kg/m^2^ | |  |  |  |  |  | 0.629 |
| Low | 19 | 0.00 (0.00,5.11) | 67 | 3.06 (0.87,10.73) | 27 | 1.80 (0.79,4.09) |  |
| Normal | 31 | 0.06 (0.00,0.74) | 122 | 2.76 (1.07,7.13) | 21 | 1.46 (0.76,2.84) |  |
| Lung cancer | |  |  |  |  |  | 0.343 |
| Yes | 11 | 0.26 (0.04,1.72) | 67 | 3.67 (1.11,12.12) | 12 | 1.97 (0.17,23.14) |  |
| No | 39 | 0.36 (0.12,1.04) | 122 | 1.59 (0.61,4.14) | 36 | 1.40 (0.81,2.41) |  |
| Digestive system cancer | | |  |  |  |  | 0.623 |
| Yes | 29 | 0.72 (0.25,2.09) | 80 | 3.55 (0.73,17.19) | 32 | 1.58 (0.72,3.46) |  |
| No | 21 | 0.34 (0.07,1.59) | 109 | 2.48 (1.02,6.02) | 16 | 2.68 (0.89,8.04) |  |
| Metastasize | |  |  |  |  |  | 0.268 |
| Yes | 12 | 0.02 (0.00,0.84) | 77 | 2.52 (1.02,6.22) | 16 | 0.75 (0.36,1.54) |  |
| No | 38 | 0.50 (0.25,1.02) | 112 | 4.05 (0.82,20.03) | 32 | 1.16 (0.58,2.35) |  |
